# Down-regulation of microRNA-216b inhibits IL-1β-induced chondrocyte injury by up-regulation of Smad3

**DOI:** 10.1042/BSR20160588

**Published:** 2017-04-28

**Authors:** Jiye He, Jiahong Zhang, Dongliang Wang

**Affiliations:** Department of Orthopaedic Surgery, Xinhua Hospital, Shanghai Jiaotong University School of Medicine, Shanghai 200092, People’s Republic of China

**Keywords:** chondrocytes, Extracellular matrix, Interleukin-1 beta, microRNA-216b, Osteoarthritis

## Abstract

Osteoarthritis (OA) is the most common type of joint disease, leading to a major cause of pain and disability. OA is characterized by the continuous degradation of articular cartilage, mainly resulting in an imbalance between synthesis and degradation of articular chondrocyte extracellular matrix (ECM). Aberrant miR-216b expression has been found in multiple cancers. However, the level of miR-216b in OA cartilage and its role in progression of this disease are still unknown. In the present study, the functional roles of miR-216b and its expression in OA tissues and interleukin-1β (IL-1β)-induced chondrocytes were examined. We found that the level of miR-216b was significantly higher and Smad3 expression was obviously lower in OA cartilage and IL-1β-induced chondrocytes than in normal tissues and cells. Furthermore, a bioinformatics analysis and luciferase reporter assay identified Smad3 as a direct target gene of miR-216b, and Smad3 expression was reduced by miR-216b overexpression at both the mRNA and protein levels. A functional analysis demonstrated that miR-216b down-regulation obviously alleviated the IL-1β-induced inhibition in cell proliferation, type II collagen, and aggrecan down-regulation and matrix metalloproteinase-13 (MMP-13) up-regulation, while miR-216b overexpression had the opposite effects. Knockdown of Smad3 by siRNA reversed the effects of the miR-216b inhibitor on cell proliferation, the expressions of type II collagen, aggrecan, and MMP-13. Our results suggested that miR-216b contributes to progression of OA by directly targeting Smad3, providing a potential therapeutic target for treatment of OA.

## Introduction

Osteoarthritis (OA) is the most common type of joint disease, leading to a major cause of pain and disability [1]. OA is characterized by the continuous degradation of articular cartilage, mainly resulting in an imbalance between synthesis and degradation of articular chondrocyte extracellular matrix (ECM) [2,3]. The important manifestation of OA is that synthesis of aggrecan and type II collagen is reduced [4,5], and production of matrix-degrading enzymes matrix metalloproteinase-13 (MMP-13) is enhanced [6]. Therefore, deep studies of OA pathogenesis are required to develop effective therapeutic strategies.

MicroRNAs (miRNAs) are short non-coding small RNAs that play essential roles in regulating diverse cellular processes such as cell proliferation, apoptosis, and differentiation [7,8]. More and more studies indicated that miRNAs are involved in the pathogenesis of OA, such as miR-26a [9], miR-142-3p [10], miR-16-5p [11], and miR-210 [12]. It has been reported that miR-216b is broadly involved in cellular processes, including proliferation, invasion, and EMT [13,14]. Aberrant miR-216b expression has been found in multiple cancers [15,16]. However, the level of miR-216b in OA cartilage and its role in progression of this disease are still unknown. It is well known that interleukin-1β (IL-1β), an inflammatory cytokine, is a major catabolic inducer and increased in OA joint tissues, contributing to the progression of OA [17,18]. IL-1β can increase production of proteases, such as matrix metalloproteinases (MMPs), and decrease synthesis of proteoglycan and collagens [19]. In addition, transforming growth factor-β (TGF-β) signaling is critical in the development, homeostasis, and repair of cartilage mainly mediated by Smad3 [20]. Chondrocytes homeostasis is crucially maintained by the balance between IL-1β and TGF-β signaling. For example, TGF-β counteracts IL-1β-induced down-regulation of ECM-related genes and up-regulation of MMP-13 [28]. On the other hand, IL-1β abrogates TGF-β-stimulated expressions of aggrecan and type II collagen [21]. We also demonstrated that the expression of Smad3 is dramatically lower in OA cartilage than in normal cartilage, which was consistent with previous reported results [22]. Given that miRNAs and Smad3 both have the important roles in the pathogenesis of OA, we explored whether miR-216b played a critical role in progression of OA by targeting Smad3.

In the present study, we found that the level of miR-216b is evidently up-regulated in OA cartilage compared with normal cartilage. Moreover, miR-216b was predicted to target the Smad3 gene and demonstrated the direct targeting of Smad3 mRNA by miR-216b. The functional analysis confirmed that miR-216b could aggravate IL-1β-induced degradation of chondrocyte ECM. Down-regulation of miR-216b increased the expressions of aggrecan, collagen type II, and Smad3, and decreased MMP-13 expression, contributing to preventing chondrocyte from IL-1β-induced ECM degradation. According to above results, miR-216b was a promising target for prevention and treatment of OA.

## Materials and methods

### Patient samples

Human cartilage samples were collected from ten OA patients undergoing total knee replacement surgery and from ten traumatic amputees without rheumatoid arthritis or OA. Patients with OA were diagnosed according to the American College of Rheumatology criteria. All samples were collected from the Department of Orthopaedic Surgery, Xinhua Hospital. Informed consent was obtained from all patients and the study was approved by the Ethics Committee of Xinhua Hospital, Shanghai Jiaotong University School of Medicine.

### Cell culture

Human chondrosarcoma cells (SW1353) have a similar phenotype to chondrocytes [22], and thus were used as substitute for cartilage cells to detect the regulatory effects of miR-216b. SW1353 cells were purchased from the A.T.C.C. (U.S.A.). SW1353 cells were cultured in DMEM with 10% fetal bovine serum (GIBCO, U.S.A.), 1% penicillin, and streptomycin (GIBCO, U.S.A.) at 37°C in a humidified 5% CO_2_ atmosphere.

Cartilage specimens were separated and cut into small pieces. Cartilage pieces for cell culture were predigested with trypsin (0.1%, w/v) (Sigma) for 30 min and collected to collagenase II (0.15%, w/v) (Sigma) digests for 16 h at 37°C. The suspension was passed through a 100-lm nylon cell strainer (BD Falcon) and rinsed in DMEM (Hyclone) with 10% FBS (GIBCO, U.S.A.). Isolated chondrocytes seeded in T-75 culture flasks in DMEM supplemented with 10% FBS (v/v), 100 units/ml penicillin, and 100 μg/ml streptomycin (GIBCO, U.S.A.). The first passage chondrocytes were used for the following experiments.

### Transient transfection

SW1353 cells were transfected with mimic and inhibitor of miR-216b (RiboBio, Guangzhou, China) at a 100 nM concentration by using Lipofectamine 3000 reagent (Invitrogen, U.S.A.) according to the manufacturer’s protocols. Following transfection, cells were stimulated with IL-1β for 24 h or were not stimulated, cell supernatants were harvested, and MMP-13 production was quantified by ELISA. Total RNA prepared from chondrocytes was used to check the expressions of miR-27b, PCNA, Smad3, MMP-13, aggrecan, and type II collagen.

### RNA extraction and quantitative real time-polymerase chain reaction

Total RNA of SW1353 cells was extracted for analyzing miRNA (Qiagen, U.S.A.) and mRNA (Axygen, U.S.A.) levels according to the manufacturer’s protocols. For quantification of miR-216b, the TaqMan MicroRNA Reverse Transcription Kit and TaqMan miRNA assay (Qiagen, U.S.A.) were used to perform reverse transcription and PCR according to the manufacturer’s instructions. U6 was used as the internal control. The gene expressions of PCNA, Smad3, MMP-13, aggrecan, and type II collagen were detected by using the SYBR Green PCR kits (TAKARA, Japan). β-Actin served as an internal control. The following primers were used: PCNA forward, 5′-CCTGCTGGGATATTAGCTCCA-3′, reverse, 5′-CAGCGGTAGGTGTCGAAGC′; Smad3 forward, 5′-CGGCTCTACTACATCGGAGG-3′, reverse, 5′-GTAGACAGCCTCAAAGCCCT-3′; MMP-13 forward, 5′-TGATGACATCAAGAAGGTGGTGAAG-3′, reverse, 5′-TCCTTGGAGGCCATGTGGGCCAT-3′; aggrecan forward, 5′-TGAGCGGCAGCACTTTGAC-3′, reverse, 5′-TGAGTACAGGAGGCTTGAGG-3′; type II collagen forward, 5′-AGAACTGGTGGAGCAGCAAGA-3′, reverse, 5′-AGCAGGCGTAGGAAGGTCAT-3′; β-actin forward, 5′-AGCGAGCATCCCCCAAAGTT-3′, reverse, 5′-GGGCACGAAGGCTCATCATT-3′; U6 forward, 5′-CGCTTCGGCAGCACATATAC-3′, reverse, 5′-AAATATGGAACGCTTCACGA-3′.

### Cell proliferation assay

ELISA-BrdU assay was performed to examine the effect of miR-216b on IL-1β-induced cell proliferation of SW1353 cells. Then, cells were seeded in 96-well plate at 5 × 10^3^ cells/well. Next, we removed the medium and transfected cells with miR-216b mimic or inhibitor at 37°C for 48 h. Finally, cells were stimulated with IL-1β for 24 h. Cell proliferation was detected by using Cell Proliferation ELISA-BrdU Kit (Millipore, U.S.A.) according to the manufacturer’s protocols.

### Western blot analysis

SW1353 cells were lysed using RIPA buffer with protease inhibitor cocktail (Millipore, U.S.A.). The protein concentration of cell lysates was quantified by BCA Kit, and 50 ng of protein was separated by SDS/PAGE (8% gel) and then transferred onto a PVDF membrane (Millipore, U.S.A.). The membranes were blocked in 5% non-fat dry milk diluted with TBST at room temperature (RT) for 1 h and probed overnight at 4°C with primary antibody, as follow: anti-Smad3 antibody (Abcam, U.S.A.). After that, the membranes were washed by TBST and incubated with a goat anti-rabbit IgG conjugated to horseradish peroxidase (Abcam, U.S.A.) for 1 h at RT. Incubation with monoclonal mouse β-actin antibody (1:10000 dilution; Sigma, U.S.A.) was performed as the loading control. The proteins were visualized using ECL Western blotting detection reagents (Millipore, U.S.A.). The densitometry of the bands was quantified using the ImageJ 1.38X software (U.S.A.).

### Dual-luciferase reporter assay

SW1353 cells were seeded in six-well plates (2 × 10^5^/well) and incubated overnight before transfection. Then, pGL3-Smad3-3′-UTR wild-type or mutant reporter plasmid, miR-216b inhibitor and anti-miR-NC, or miR-216b mimic and miR-NC, and pRL-TK Renilla luciferase reporter (Promega, U.S.A.) were cotransfected into cells by using Lipofectamine 3000 (Invitrogen, U.S.A.). After that, luciferase activities were quantified using the Dual-Luciferase reporter system (Promega, U.S.A.) according to the manufacturer’s protocols. Firefly luciferase activities were normalized to renilla luciferase activities.

### MMP-13 enzyme-linked immunosorbent assay

After transfection, SW1353 cells were stimulated with IL-1β (5 ng/ml) for 24 h, and the MMP-13 protein in the culture supernatants was quantified by using an ELISA kit following the manufacturer’s protocol (R༆D, U.S.A.). Plates were read at 450 nm using a Microplate Reader (Thermo, U.S.A.), and the MMP-13 concentration in the samples was calculated using a standard curve.

### Statistical analysis

Experiments were repeated at least three times. Values are expressed as mean ± standard error of the mean (S.E.M.). Data were evaluated for statistical significance by analysis using one-way analysis of variance (ANOVA). *P*<0.05 was considered to indicate a statistically significant difference. All statistical analyses were performed using GraphPad Prism 5.0 (GraphPad Software, Inc., U.S.A.).

## Results

### miR-216b level was up-regulated and Smad3 expression was down-regulated in OA cartilage and IL-1β-induced SW1353 cells

**Figure 1:**
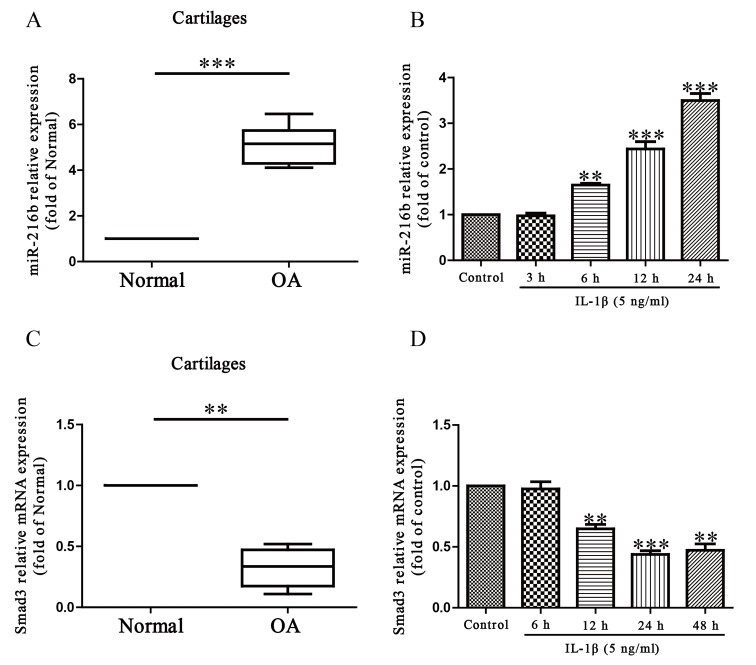
The level of miR-216b in OA cartilage and IL-1β-induced SW1353 cells (**A**) Relative expression of miR-216b in normal (*n*=10) and OA cartilages (*n*=10) was detected by qRT-PCR. (**B**) SW1353 cells were treated with 5 ng/ml IL-1β for 0, 3, 6, 12, or 24 h. Level of miR-216b was analyzed by qRT-PCR and normalized to U6. (**C**) Relative expression of Smad3 in normal (*n*=10) and OA cartilages (*n*=10) was detected by qRT-PCR. (**D**) SW1353 cells were treated with 5 ng/ml IL-1β for 0, 6, 12, 24, or 48 h. Level of Smad3 was analyzed by qRT-PCR and normalized to β-actin. The data shown are mean ± S.E.M., *n*=4; **P<0.01, ***P<0.001 versus Normal or Control.

To study the possible role of miR-216b in the OA processes, we determined its expression level by quantitative real time-polymerase chain reaction (qRT-PCR) in normal and OA articular cartilage. Our results demonstrated that the level of miR-216b was dramatically increased in OA articular cartilages compared with that in normal groups (Figure 1A). Next, we studied the level of miR-216b in IL-1β-induced SW1353 cells. As expected, SW1353 cells were treated with IL-1β at 5 ng/ml for 0, 3, 6, 12, or 24 h, after which the levels of miR-216b were detected. Compared with untreated controls, our findings showed that IL-1β could significantly increase the level of miR-216b in a time-dependent manner (Figure 1B). Subsequently, the online database, TargetScan 6.2, showed that Smad3 was predicted to be a direct target of miR-216b. Then, we tested the mRNA level of Smad3 in OA cartilage and IL-1β-induced SW1353 cells respectively. The results indicated that the expression of Smad3 was significantly decreased in OA cartilage and IL-1β-induced SW1353 cells at mRNA level (Figure 1C and D). From above outcomes, we predicted that miR-216b might negatively regulate Smad3.

### Effects of miR-216b on IL-1β-stimulated proliferation of primary chondrocytes and SW1353 cells

**Figure 2:**
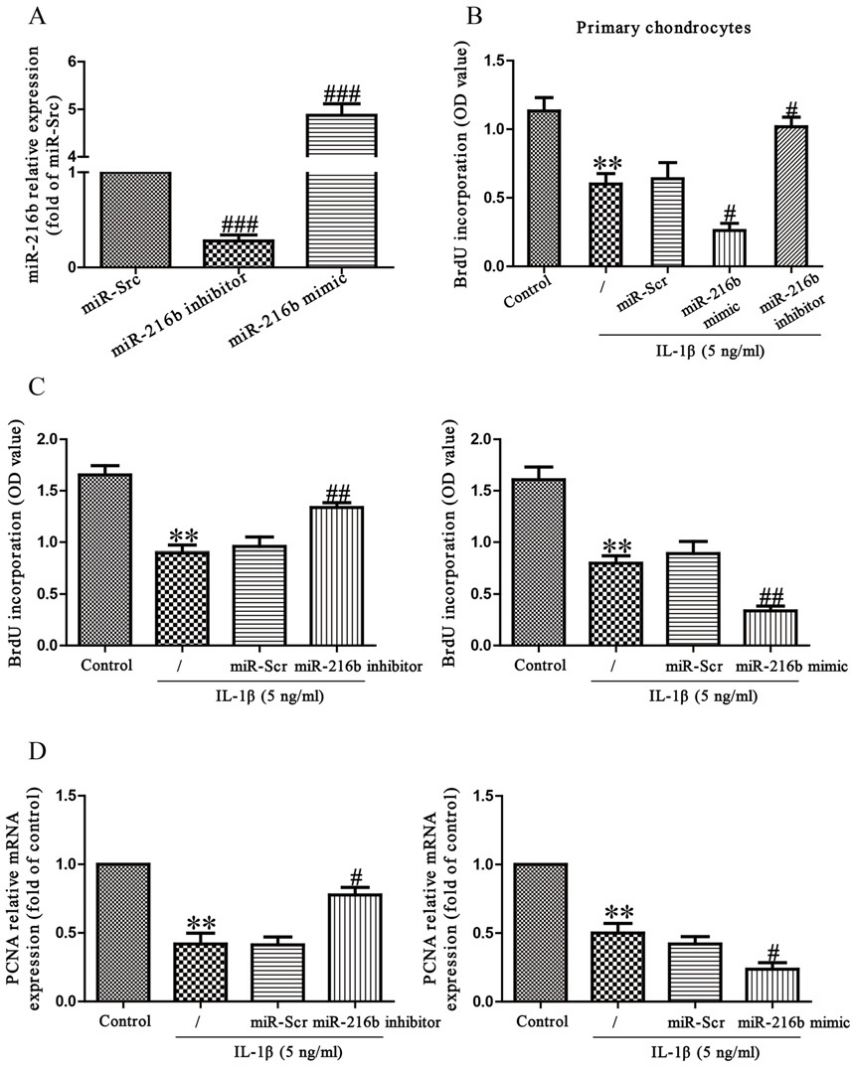
Effects of miR-216b on IL-1β-induced cell proliferation in primary chondrocytes and SW1353 cells Primary chondrocytes and SW1353 cells were transfected with miR-216b mimic or inhibitor. (**A**) The level of miR-216b in SW1353 cells was determined by RT-PCR. SW1353 cells were treated with IL-1β (5 ng/ml) for 24 h after transfection with miR-216b mimic or inhibitor. (**B**) and (**C**) Cell proliferation was assessed by BrdU-ELISA assay. (**D**) The mRNA expression of PCNA was determined by qRT-PCR. β-Actin was detected as a loading control. All data are presented as mean ± S.E.M., *n*=6; **P<0.01 versus Control, ^#^*P*<0.05, ^##^*P*<0.01, ^###^*P*<0.001 versus miR-Scr.

To study the miR-216b impact on the IL-1β-induced chondrocytes, we transfected either miR-216b mimic or inhibitor into the primary chondrocytes and SW1353 cells and then stimulated them with IL-1β. First, we evaluated the miRNA transfection efficiency via the RT-PCR assay. After transfection for 48 h, as shown in Figure 2A, the level of miR-216b was obviously increased in the miR-216b mimic group compared with the miR-Scr group. Then, the miR-216b level was significantly decreased in SW1353 cells transfected with miR-216b inhibitor compared with the cell treated with miR-Scr. To determine the role of miR-216b in proliferation of primary chondrocytes and SW1353 cells, the results from Brdu-ELISA assay demonstrated that knockdown of miR-216b dramatically promoted the proliferation of primary chondrocytes and SW1353 cells stimulated by IL-1β, whereas overexpression of miR-216b significantly inhibited IL-1β-induced proliferation of primary chondrocytes and SW1353 cells (Figure 2B and C). To further confirm this result, we detected the expression of PCNA mRNA. We found that miR-216b inhibitor could evidently increase the expression of PCNA compared with IL-1β group, and miR-216b mimic further decreased IL-1β-stimulated expression of PCNA (Figure 2C).

### Effects of miR-216b on IL-1β-induced MMP-13 production in SW1353 cells

**Figure 3:**
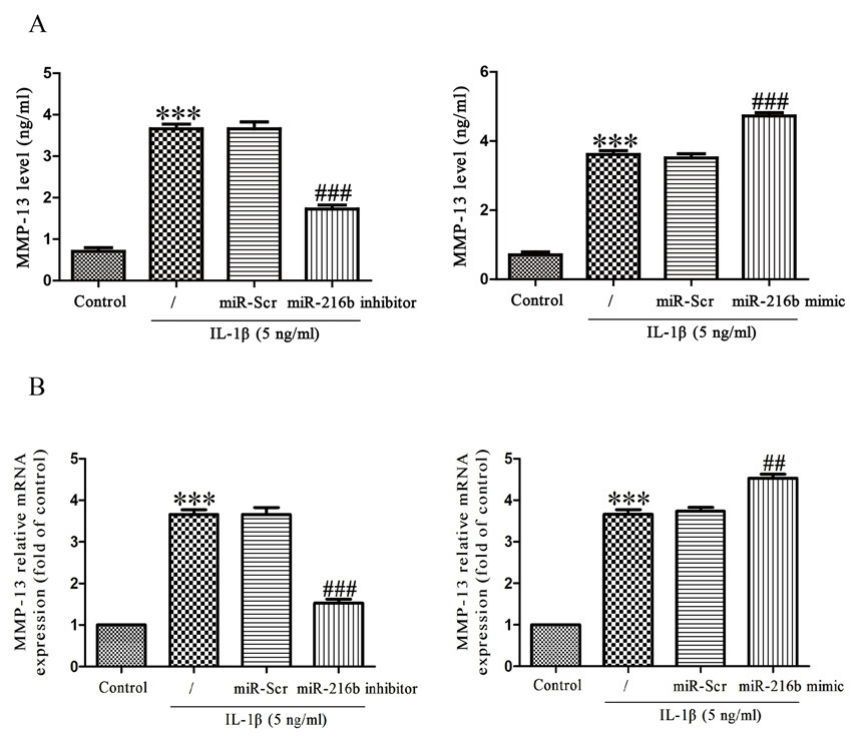
Effect of miR-216b on IL-1β-induced MMP-13 in SW1353 cells SW1353 cells were treated with IL-1β (5 ng/ml) for 24 h after transfection with miR-216b mimic or inhibitor. (**A**) The expression level of secreted MMP-13 protein was determined by ELISA. (**B**) The mRNA level of MMP-13 was determined by qRT-PCR. The data shown are mean ± S.E.M., *n*=4; ***P<0.001 versus Control, ^##^*P*<0.01, ^###^*P*<0.001 versus vehicle + IL-1β.

MMP-13, as the major collagenase contributing to the catabolic processes in OA, cleaves key ECM constituents including type II collagen and aggrecan. We treated SW1353 cells with IL-1β (5 ng/ml) for 24 h after transfection with miR-216b mimic or inhibitor, and then determined MMP-13 expression at mRNA and protein levels by qRT-PCR and MMP-13 ELISA respectively. IL-1β stimulation of chondrocytes resulted in the secretion of MMP-13 protein in the culture supernatant, and then down-regulation of miR-216b could significantly reduce the secretion of MMP-13 induced by IL-1β (Figure 3A). Next, down-regulation of miR-216b evidently inhibited the increase in IL-1β-induced MMP-13 expression at mRNA level (Figure 3B). On the contrary, overexpression of miR-216b could promote IL-1β-stimulated expression of MMP-13 (Figure 3A and B).

### Effects of miR-216b on IL-1β-induced inhibition of aggrecan and type II collagen synthesis in SW1353 cells

**Figure 4:**
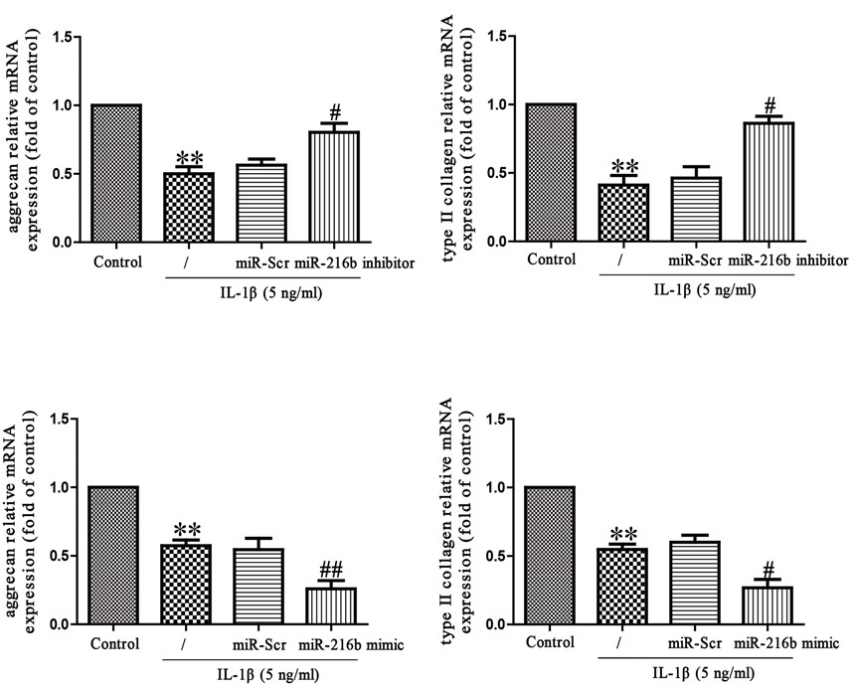
Effects of miR-216 on IL-1β-induced inhibition of aggrecan and type II collagen synthesis in SW1353 cells SW1353 cells were treated with IL-1β (5 ng/ml) for 24 h after transfection with miR-216b mimic or inhibitor. The mRNA levels of aggrecan and type II collagen were determined by qRT-PCR. The data shown are mean ± S.E.M., *n*=4; **P<0.01 versus Control, ^#^*P*<0.05, ^##^*P*<0.01 versus vehicle + IL-1β.

To explore the effect of miR-216b on IL-1β-induced matrix degradation, we transfected miR-216b mimic or inhibitor into SW1353 cells that were exposed to IL-1β for 24 h, and then mRNA levels of aggrecan and type II collagen were analyzed by qRT-PCR analysis. As expected, mRNA levels of aggrecan and type II collagen were significantly restored in SW1353 cells transfected with miR-216b inhibitor when compared with IL-1β-treated SW1353 cells (Figure 4). However, miR-216b mimic specifically reduced IL-1β-induced aggrecan and type II collagen synthesis (Figure 4), further confirming the effect of miR-216b in aggrecan and type II collagen synthesis.

### miR-216b can directly target Smad3 in SW1353 cells

**Figure 5:**
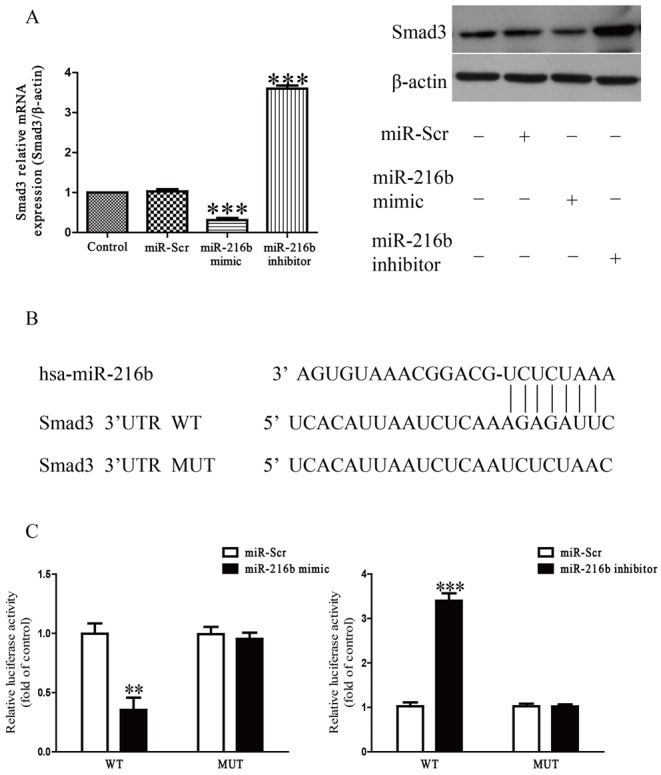
Smad3 was a direct target of miR-216b SW1353 cells were treated with IL-1β (5 ng/ml) for 24 h after transfection with miR-216b mimic or inhibitor. (**A**) The mRNA and protein levels of Smad3 were determined by qRT-PCR or Western blot respectively. Smad3 expression was normalized to β-actin. (**B**) Schematic representation of Smad3 3′-UTRs showing putative miRNA target site. (**C**) The analysis of the relative luciferase activities of Smad3-WT and Smad3-MUT. All data are presented as mean ± S.E.M., *n*=4; **P<0.01, ***P<0.001 versus Control or miR-Scr.

We used the online database (TargetScan 6.2) to predict the target gene, and found that Smad3 was a binding target of miR-216b. We performed qRT-PCR to detect the expression of Smad3 at mRNA and protein levels in SW1353 cells transfected with miR-216b inhibitor or mimic. Results indicated that mRNA and protein levels of Smad3 were dramatically down-regulated after overexpression of miR-216b (Figure 5A), but were significantly up-regulated after inhibition of miR-216b compared with control group (Figure 5A). To further confirm whether Smad3 was a direct target of miR-216b, we constructed luciferase reporter vector containing the WT 3′-UTR Smad3, and the putative miR-216b-binding site in the Smad3 3′-UTR was mutated (MUT 3’-UTR Smad3) (Figure 5B). Our findings showed that miR-216b mimic or inhibitor significantly suppressed or promoted the luciferase activity in SW1353 cells transfected with WT 3′-UTR of Smad3 respectively (Figure 5C). The MUT Smad3 3′-UTR abolished the effect of miR-216b. These results suggested that Smad3 was directly and negatively regulated by miR-216b, and miR-216b might function in OA by regulation of Smad3.

### Inhibition of Smad3 is essential for protective effect of miR-216b on IL-1β-induced chondrocyte injury in SW1353 cells

**Figure 6:**
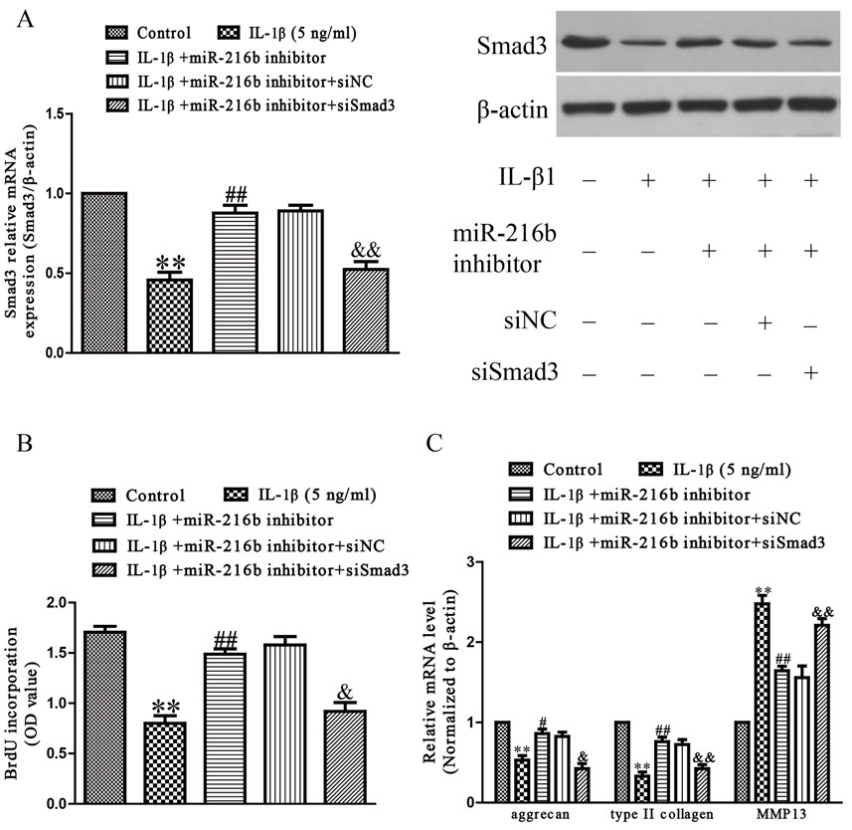
Smad3 was involved in the effects of miR-216b on IL-1β-induced cartilage degradation in SW1353 cells SW1353 cells were transfected with either miR-216b inhibitor or si-Smad3, and then treated with IL-1β (5 ng/ml) for 24 h. (**A**) The mRNA and protein levels of Smad3 were determined by qRT-PCR or Western blot respectively. Smad3 expression was normalized to β-actin. (**B**) Cell proliferation was assessed by BrdU-ELISA assay. (**C**) The mRNA levels of aggrecan, type II collagen, and MMP-13 were determined by qRT-PCR. β-Actin was detected as a loading control. All data are presented as mean ± S.E.M., *n*=4; **P<0.01 versus Control, ^#^*P*<0.05, ^##^*P*<0.01 versus vehicle + IL-1β, ^&^*P*<0.05, ^&&^*P*<0.01 versus IL-1β + miR-216b inhibitor.

Next, to explore whether miR-216b inhibition protected IL-1β-induced SW1353 cells in an Smad3-dependent manner, we cotransfected SW1353 cells with miR-216b inhibitor and si-Smad3, and then stimulated with IL-1β. We found that the expression of Smad3 was significantly increased after transfection with miR-216b inhibitor in IL-1β-induced SW1353 cells compared with IL-1β treatment group, and down-regulation of Smad3 by siRNA could block the effect of miR-216b inhibitor on Smad3 expression (Figure 6A). The effect that silence of miR-216b attenuates IL-1β-induced inhibition of SW1353 cell proliferation (Figure 6B), down-regulation of aggrecan, type II collagen, and up-regulation of MMP-13 mRNA expression were blocked by added si-Smad3 (Figure 6C). From all above results, we clearly demonstrated that down-regulation of miR-216b improved IL-1β-induced increases in cell differentiation and collagen synthesis of SW1353 cells by up-regulation of Smad3, and that up-regulation of Smad3 was essential for the protective effect of miR-216b inhibition on IL-1β-induced cardiac fibrosis in SW1353 cells.

## Discussion

Dysregulation of miRNA levels is closely related to multiple diseases, including OA [23,24]. The up-regulation of miR-377, miR-103, miR-483, and miR-22, as well as the down-regulation of miR-25, miR-26a, miR-337, and miR-299 have been detected in OA cartilage compared with normal cartilage [25]. In addition, many miRNAs have known roles in the regulation of OA progression. For example, microRNA-142-3p inhibits chondrocyte apoptosis and inflammation in OA by inhibiting the HMGB1-mediated NF-κB signaling pathway [10]. miR-210 inhibits NF-κB signaling pathway by targeting DR6 in OA [12]. Therefore, it is critical to characterize the gene networks regulated by these miRNAs in OA. In the present study, we demonstrated that the level of miR-216b is significantly up-regulated in OA cartilage compared with normal cartilage, and also in response to IL-1β stimulation. It suggested that miR-216b is potentially involved in pathogenesis of OA.

IL-1β has a critical role in cartilage degradation during development of OA [18]. Articular cartilage ECM regulates many biological processes including chondrocyte growth, differentiation, attachment, and survival, which are important for the repair and homeostasis of cartilage [26]. The continuous loss of cartilage ECM impairs joint function and is involved in the progression of OA [4]. Many studies have demonstrated that IL-1β greatly regulates the production of chondrocytes ECM components such as aggrecan and collagen type II [27]. Besides, IL-1β influences the synthesis of the MMPs in the chondrocytes, mainly MMP-1, MMP-3, and MMP-13, which have a destructive effect on the cartilage components [28]. Consistent with previous studies, we found that IL-1β significantly down-regulated expressions of aggrecan and collagen type II in SW1353 cells. We performed a functional analysis of miR-216b to determine whether it regulated the expressions of the ECM components MMP-13, type II collagen, and aggrecan. Modulation of miR-216b efficiently affected IL-1β-stimulated degradation and synthesis of ECM in OA chondrocytes, as evidenced by that inhibition of miR-216b was able to reverse IL-1β-induced impairment of aggrecan, type II collagen, and evidently inhibited the increase in IL-1β-induced MMP-13 expression. Conversely, overexpression of miR-216b aggravated IL-1β-induced down-regulation of aggrecan, type II collagen, and further increase IL-1β-induced production of MMP-13. These results indicated that the down-regulation of miR-216b inhibited the progression of OA by promoting chondrocyte ECM synthesis.

Furthermore, we identified that miR-216b directly regulated Smad3 that was an essential factor for chondrocyte homeostasis by binding the predicted seed sites, contributing to the inhibition of Smad3 expression at both mRNA and protein levels. Besides, we observed an inverse correlation between Smad3 and miR-216b expression in OA cartilage and chondrocytes treated with IL-1β. We found that the 3′-UTR of Smad3 contained a binding site of miR-216b, and Smad3 expression was obviously reduced in OA cartilage compared with normal cartilage. Smad3 also plays a critical role in mediating TGF-β signal transduction [29]. Many reports have demonstrated the critical role of TGF-β/Smad3 signaling in maintaining articular cartilage and preventing OA [20,30]. OA is characterized by impairment of TGF-β signals, which attributes to Smad3 disruption [30]. Moreover, mutation of Smad3 leads to cartilage degeneration and OA. Smad3 regulates the balance between the synthesis and degradation of ECM of articular chondrocyte by decreasing expression of MMP-13 and increasing expressions of aggrecan and type II collagen [31]. Our data showed that miR-216b directly binds to the 3′-UTR of Smad3. Additionally, down-regulation of miR-216b markedly up-regulated the mRNA and protein expressions of Smad3. However, overexpression of miR-216b apparently decreased the mRNA and protein expressions of Smad3. These results demonstrated that miR-216b might play a crucial role in controlling the development of OA by directly targeting Smad3. Consequently, we speculated that down-regulated miR-216b inhibited the progression of OA via up-regulation of Smad3. Next, we confirmed that the up-regulation of aggrecan and type II collagen expression, which was induced by the inhibition of miR-216b, was significantly reduced by down-regulation of Smad3. Our results suggested that the increased miR-216b expression inhibited Smad3 expression, thereby suppressing ECM synthesis of chondrocyte and contributing to the progression of OA.

In conclusion, our findings indicated that miR-216b down-regulation directly increased the expression of Smad3, contributing to protecting against IL-1β-induced chondrocytes ECM degradation. Further studies of miR-216b may present new insights into disease mechanisms and treatment in OA.

## Abbreviations

ECM: extracellular matrix
IL-1β: interleukin-1β
MMP: matrix metalloproteinase
OA: osteoarthritis
qRT-PCR: quantitative real time-polymerase chain reaction
RT: room temperature
TGF-β: transforming growth factor-β
